# Reconstruction left atrium and isolation pulmonary veins of paroxysmal atrial fibrillation using single contact force catheter with zero x-ray exposure: A CONSORT Study

**DOI:** 10.1097/MD.0000000000007726

**Published:** 2017-10-13

**Authors:** Jian Qiang Zhang, Rong Hui Yu, Jia Bing Liang, De Yong Long, Cai Hua Sang, Chang Sheng Ma, Jian Zeng Dong

**Affiliations:** a Department of Cardiovascular Medicine, Shanghai East Hospital, Tongji University School of Medicine, Shanghai; b Beijing Anzhen Hospital, Capital Medical University, Beijing; c Juxian People Hospital, Shandong Province 276500,China.

**Keywords:** atrial fibrillation ablation, reconstruction left atrium, single catheter, zero x-ray exposure

## Abstract

**Background:**

Conventional ablation of paroxysmal atrial fibrillation (PAF) is associated with radiation risks for patients and laboratory staff. Three-dimensional (3D) mapping system capable of showing contact force (CF) and direction of catheter tip may compensate for nonfluoroscopic safety issues.

**Objective:**

The aim of this study was to investigate the feasibility of zero x-ray exposure during reconstruction left atrium (LA) and ablation.

**Methods:**

Single, CF catheter, and 3D mapping system were used to reconstruct LA and isolate pulmonary veins (PV) in all patients. The patients were randomly divided into 2 groups after LA angiography. In group 1, reconstruction LA and isolation PV was performed with the help of 3D system (without x-ray), whereas in group 2, x-ray and 3D system were utilized to reconstruct LA and ablate PV antrum. After ablation, Lasso catheter was used to confirm the PV isolation. All patients were followed up to 12 months.

**Results:**

A total of 342 PAF patients were continuously enrolled. The basic clinical characteristics between the 2 groups had no significant difference. Parameters related to the procedure, average procedure time, ablation procedure time, average contact force (CF) applied, the percentage of time within CF settings, and average power applied during radiofrequency application showed no significant difference between the 2 groups. In group 1, the average fluoroscopy time before LA reconstruction was similar to that in group 2 (2.8 ± 0.4 vs. 2.4 ± 0.6 minutes, *P* = .75). The average fluoroscopy time during ablation was significantly lower than that in group 2 (0 vs. 7.6 ± 1.3 minutes, *P* < .001). The total x-ray exposure dose of the procedure in group 1 was significantly lower than that in group 2 (19.6 ± 9.4 vs. 128.7 ± 62.5 mGy, respectively, *P* < .001). Kaplan-Meier analysis indicated that there were no statistical differences in the probability of freedom from atrial arrhythmia (AF/AFL/AT) recurrence at 12 months between group 1 and group 2 (*P* = .152). The success rate after a single ablation procedure and without drugs (Class I/III AAD) at 12 months was not significantly different between the 2 groups (67.6%, 95% confidence interval [CI]: 62%–79.5% in group 1 and 68.9%, 95% CI: 63%–80.7% in group 2, *P* = .207). Procedural-related adverse events showed no significant different incidence between group 1 and group 2. A multivariate logistic regression analysis of risk factors was performed to evaluate the effectiveness outcome, which demonstrated that the percentage of CF (within the investigator-selected work ranges) during therapy was significantly associated with positive outcomes (odds ratio: 3.68; 95% CI: 1.65–10.6, *P* = .008), whereas the LA dimension was negatively associated with effectiveness outcomes (odds ratio: 0.72; 95% CI: 0.52–0.84, *P* = .016).

**Conclusions:**

Reconstruction LA and isolation PV ablation using single CF-assisted catheter without x-ray exposure was both safe and effective. CF was positively associated with effective outcomes and LA dimensions negatively with effective ones.

## 1 Introduction

Radiofrequency catheter ablation (RFCA) therapy is an important treatment for paroxysmal atrial fibrillation (PAF)^[1]^ and is considered to be a first-line rhythm-control strategy in patients without any structural heart disease.^[2]^ The catheter is usually guided by fluoroscopy, which often results in both patients and medical staff being exposed to significant levels of radiation.

Three-dimensional (3D) mapping systems (e.g., CARTO 3 system) can significantly reduce operation time and x-ray exposure.^[3]^ The system can, in a timely fashion, provide the direction and location of the catheter apex on a screen. The apex of the catheter showed the “drop-off” motion when it moved from inside the pulmonary vein (PV) to the left atrium (LA), indicating the location of the PV antrum, which is the target of RFCA. This demonstrates that the ablation target can be identified without x-rays, thus allowing operators to reconstruct LA and isolate PV with little or no x-ray exposure, which is termed “green ablation.” Recent clinical studies have suggested that using a CF-sensing catheter improves the efficiency of ablation by providing the force and direction of the contact apex in PAF patients.^[4]^

This study was designed to evaluate, in a randomized fashion, the safety and effectiveness of zero x-ray exposure to reconstruct LA and ablate PAF after LA angiography.

## 2 Methods

PAF patients were continuously enrolled at the Anzhen Hospital (Beijing, China). The institutional review board approved the study protocol and all study participants provided written informed consent. Enrollment required at least 3 symptomatic AF episodes within the 6 months before enrollment, and nonresponse to at least 1 antiarrhythmic drug (Class I or Class III).

Patients were treated routinely with antiarrhythmic medication before discharge and the medication was typically discontinued after 12 weeks. The patients were clinically evaluated at 3, 6, and 12 months, during which they were queried for symptoms and 12-lead electrocardiograms (ECG) were obtained. AF recurrence was defined as a self-reported symptomatic episode of arrhythmia with ECG documentation or as an asymptomatic AF detected by 24-hour Holter recording.

Criteria for exclusion from the study were as follows: patients <18 years of age, ejection fraction <40%, documented LA thrombus, coronary artery bypass graft procedure in the previous 6 months, New York Heart Association functional class III or class IV, myocardial infarction within the previous 2 months, a thromboembolic event in the previous 12 months, severe pulmonary disease, previous valvular cardiac surgical procedure, and life expectancy of <12 months.

After atrial septal puncture, left and right PV angiographies were performed in the right anterior oblique (RAO) at a 30-degree angle. The patients were randomly divided into 2 groups before LA reconstruction. In group 1, only CARTO 3 system was used to construct a virtual LA shell and isolate PV (without x-ray), whereas in group 2, x-ray and CARTO 3 system were utilized to reconstruct LA and isolate PV.

Reconstruction LA 3 rings were the emphasis to be rebuilt, mitral valve ring and two rings along left and right PV antrum, using two complementary positions on the CARTO screen RAO and left anterior oblique. Mitral valve ring was reconstructed according to the local potential of small “A” (representing atrium) and big “V” (representing ventricle). The two rings along PV antrum were rebuilt according to 3 characteristics, local potential change, impedance reduction, and drop-off motion. When the catheter apex was moved from inside of the PV to the LA, the local potential shifted from a single, shaped wave to fused wave and local impedance was reduced. The apex of catheter was also able to show a unique drop-off motion that can be found within some special combination zones of the LA and PVs, such as the anterior and inferior sides of right PV and the front or inferior side of left PV. Combining these 3 characteristics, with particular emphasis on the noted drop-off motion, the location of PV antrum can be positively determined. The ablation catheter has been described previously in detail.^[5]^ PV isolation (PVI) with confirmation of entrance blockage was required. After ablation, CF catheter was replaced by Lasso catheter to confirm the PVI.

PVs were isolated using a circumferential lesion kit. In the event of spontaneous atrial flutter (AFL), additional ablation was performed at the investigator's discretion, and included LA linear lesions (mitral isthmus line between the mitral annulus and left inferior PV or cavotricuspid isthmus line between the inferior vena cava and cavotricuspid annulus). Ablation was considered to be finished when the line was blocked in both directions. Following ablation, anticoagulation with warfarin was required for the first 3 months.

A Thermocool SmartTouch catheter (Biosense Webster, Inc., Diamond Bar, CA) was used for ablation. The power was based on the location of radiofrequency ablation, rather than CF, and it was limited to 35 W at the anterior, superior, and inferior sites (flow rate, 17 mL/min) and 25 W at the posterior wall (flow rate, 17 mL/min). The temperature limit was 43°C and the time limit was 30 seconds for each lesion. All patients received heparin intravenously (activated clotting time >300 seconds) to avoid thromboembolic complications.

The value of CF was obtained successfully while manipulating the catheter to assess catheter-tissue contact. The vector position was manipulated visually and this aided the operators in both obtaining CF values and precisely controlling the catheter position. A working range of 5 to 30 g was selected for all study procedures. Any time the CF is <5 or >30 g, ablation should be stopped. The average CFs used during radiofrequency application to the posterior and upper wall were between 5 and 10 g, whereas other procedures on the inferior and anterior wall had an average CF range between 10 and 30 g.

The CF data for all ablation sites used to achieve PVI were recorded and analyzed offline after the procedure. During the procedure, CARTO3 console continuously estimated the value of CF once every 50 ms. The average values during the previous 1000 ms were displayed and updated in real time. During ablation, as each data point was measured and collected, the respective CF value (mean of the measurements in the previous 1000 ms) was recorded. These values were used to estimate the average CF and standard deviation.

The procedure resulted in reduced documented symptomatic AF/atrial tachycardia (AT)/AFL during the 12-mo follow-up. AF/AT/AFL episodes of >30 seconds were considered to be recurrent events, whereas major adverse events were defined as procedure- or device-related serious adverse events (e.g., death, stroke, tamponade, pericarditis, pericardial effusion, perforation), which occurred within 7 days following the ablation procedure. Atrioesophageal fistula that occurred within 40 days post-procedure was also included.

### 2.1 Statistical analysis

Continuous parameters were expressed as mean ± standard deviation. Procedural data were compared by an unpaired *t* test. Categorical values were compared using a *χ*^2^ test. The Pearson correlation coefficient was used to evaluate the correlation between the continuous variables. A multiple logistic regression analysis was used to determine which baseline clinical, echocardiographic, or therapeutic variables predicted postablation AF recurrence at the 12-month follow-up. *P* values <.05 were considered to be significant. Statistical analyses were performed using with the Statistical Package for Social Sciences (SPSS Inc, Cary, NC).

## 3 Results

Between July 7, 2015 and January 28, 2016, the study enrolled 342 patients. The patients were randomly divided into 2 groups. In group1, zero x-ray exposure was used in reconstruction LA and ablation procedure, which included 170 patients, whereas in the remaining 172 patients, comprising group 2, x-ray was used to reconstruct LA and isolate PV**.** Six patients of group 2 were lost to follow-up before reaching the 12-month follow-up visit. Patient characteristics are described in Table 1.

**Table 1:**
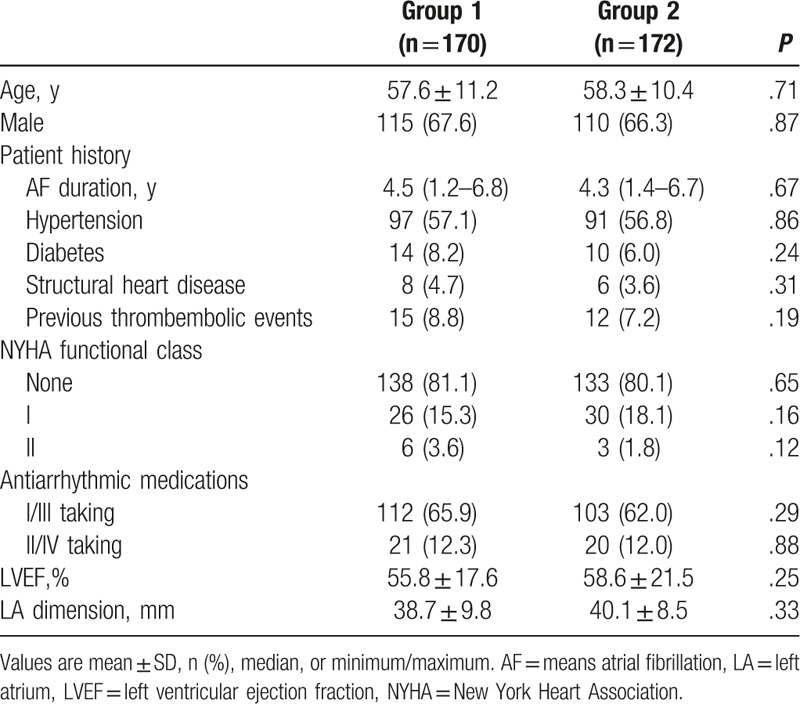
Baseline patients characteristics.

**Figure 1:**
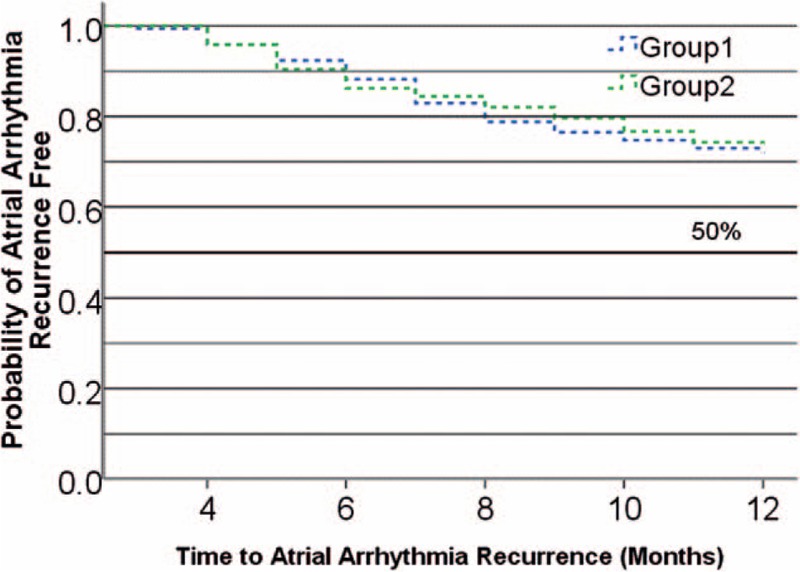
Kaplan-Meier curve of time to atrial arrhythmia recurrence probability of freedom from symptomatic atrial arrhythmia recurrence at 12-month follow-up visit.

Among the 2 groups (Table 2), the average procedure time (65 ± 24 in group 1 and 63 ± 26 minutes in group 2, *P* = .45), ablation time (38 ± 18 in group 1 and 42 ± 16 minutes in group 2, *P* = .12), and the average power applied during radiofrequency application (30.8 ± 6.8 in group 1 and 29.8 ± 5.7 W in group 2, *P* = .34) were not significantly different. The overall average CF recorded at every point during ablation indicated that there were no significant differences between the 2 groups (13.8 ± 7.5 in group 1 and 15.2 ± 8.3 g in group 2, *P* = 0.11). The percentage of time within CF settings showed no significant differences between the 2 groups (95% ± 4.2% in group 1 and 96% ± 3.8% in group 2, *P* = .43). In addition, the rate of additional line ablation performed between 2 groups also did not demonstrate significant differences (Table 2). In group 1, the average fluoroscopy time before ablation was similar to that in group 2 (2.8 ± 0.4 vs. 2.4 ± 0.6 minutes, *P* = 0.71). The average fluoroscopy time during LA reconstruction and ablation was significantly lower than in group 2 (0 vs. 7.6 ± 1.3 minutes, *P* < .001). The total x-ray exposure dose of the procedure in group 1 was significantly lower than that in group 2 (19.6 ± 9.4 vs. 128.7 ± 62.5 mGy, *P* < .001). Kaplan-Meier analysis indicated that there were no statistical differences in the probability of freedom from atrial arrhythmia (AF/AFL/AT) recurrence at 12 months between group 1 and group 2 (*P* = .152, Fig. 1). The success rate after a single ablation procedure and without drugs (Class I/III AAD) at 12 months was not significantly different between the 2 groups (67.6%, 95% confidence interval [CI]: 62%–79.5% in group 1 and 68.9%, 95% CI: 63%–80.7% in group 2, *P* = .207).

**Table 2:**
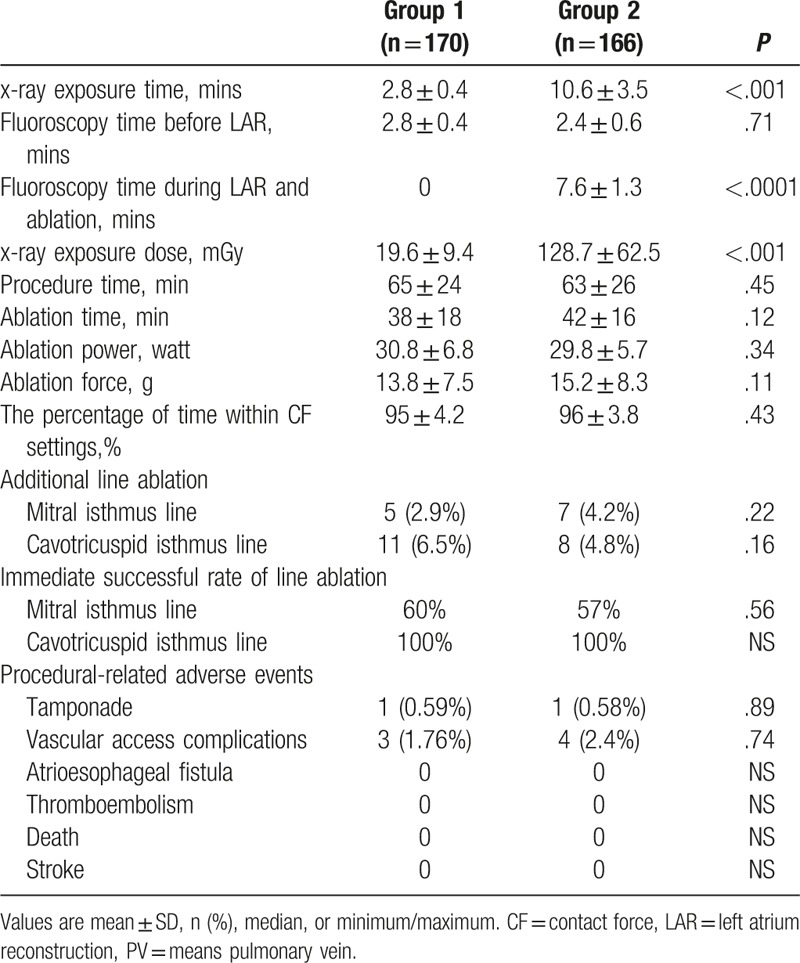
Results of parameters related to ablation between the 2 groups.

Many factors that are indicative of risk in effectiveness were identified by univariate analysis. This included the LA dimension, LVEF, AF burden, age, blood pressure, percentage of time with CF within investigator selected, and x-ray exposure time. Using all parameters deemed to be significant by univariate analysis, a multivariate logistic regression analysis of risk factors was performed to evaluate the effectiveness outcome (Table 3), which demonstrated that the percentage of CF (within the investigator-selected work ranges) during therapy was significantly associated with positive outcomes (odds ratio:3.68; 95% CI: 1.65–10.6, *P* = .008), whereas the LA dimension was negatively associated with effectiveness outcomes (odds ratio:0.72; 95% CI: 0.52–0.84, *P* = .016).

**Table 3:**
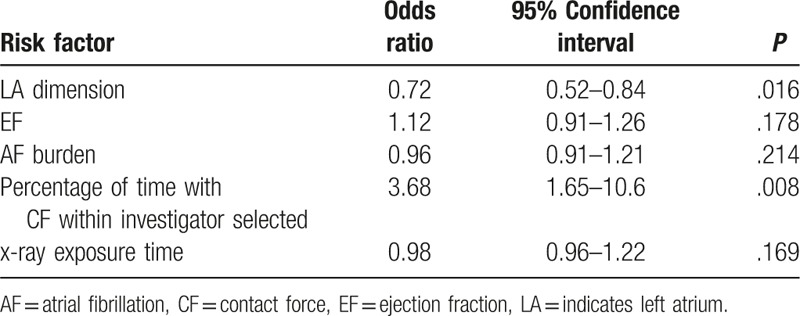
Multivariate logistic regression analysis of risk factors for primary effectiveness endpoint (n = 342).

No deaths, strokes, cerebrovascular accidents, atrioesophageal fistula, myocardial infarctions, or thromboembolisms occurred within the study period. Procedure-related adverse events occurred within 7 days of the ablation procedure that was observed in 9 (2.6%) patients, including 2 (0.58%) tamponades and 7 (2.1%) vascular access complications, which did not demonstrate significant differences between groups 1 and 2 (Table 2).

## 4 Discussion

The results of this study suggested that single catheter reconstruction LA and isolation PV without x-ray exposure is safe and effective when paired with a 3D system and a CF-assisted catheter. CF and LA dimensions were associated with positive outcomes.

### 4.1 Radiation risks with PAF ablation

Radiation exposure to patients and medical staff should be considered during PAF ablation. The procedure still requires a relatively long exposure to radiation during ablations^[6]^ and obese patients are at particular risk.^[7]^ The predicted excess risk of fatal malignancies is 0.07% in women and 0.1% in men.^[6]^ Moreover, approximately one-third of AF patients require repeated ablation procedures. Thus, reduced fluoroscopic times are necessary in protecting AF patients.

Medical laboratory staffs may be at even greater risk. Fluoroscopy time for catheter ablation of AF varied widely in published studies, from 148 to 6 minutes.^[8]^ Using advanced technology, the potential for further decreasing radiation exposure has increased.^[9,10]^ The major reduction in radiation exposure relies on shielded console, yet this only minimizes fluoroscopy during ablation. In this study, the average fluoroscopy time per procedure was ∼2.8 minutes in group 1, which was shorter than the most minimal reported time (6 ± 2 minutes) from a study using intracardiac ultrasound echocardiography and 3D system.^[8,11]^ The total x-ray exposure was significantly reduced by ∼19.6 mGy in group 1. The variation in exposure times likely reflects variations in experience and technique, whereas green ablation reflects the operator's knowledge of LA anatomy. These modifications to the procedure could greatly reduce the fluoroscopy time and dose, which will protect physicians.

### 4.2 The 3D system and CF catheter improves the safety of the ablation procedure

Previous studies showed that low CF is associated with ineffective lesion during ablation.^[12,13]^ Although electrogram amplitude and local impedance can be used to estimate CF, there is a poor relationship between CF and the unipolar or bipolar atrial potential amplitude/impedance.^[14]^ Increasing the CF produced a progressive increase in lesion depth for constant radiofrequency power, whereas the incidence of steam pop and thrombus formation also increases with increasing CF.^[15–17]^ In the present study, the radiofrequency was modulated for ∼30 W with a CF of 15 g. Acute PV antrum isolation was achieved in all patients (median 38 minutes), despite decreased CF at high radiofrequency power. Multivariate logistic regression analyses indicated that CF was significantly associated with positive outcomes (odds ratio: 3.68), suggesting that this methodology was safe for the study population under the specific ablation conditions (power, 30 W; CF, 15 g). Importantly, when steamed pop occurred during isolation of the cavotricuspid isthmus, the most frequent location of steam pop, tamponades incidence was rare. However, 2 tamponades events did occur when the average CF was >30 g during ablation of mitral isthmus line. The patients recovered soon after pericardiocentesis and were safely discharged several days later.

3D system integrated with CF catheter is useful in visualizing the catheter direction in real-time with zero-fluoroscopy ablation of right and left atrial arrhythmia.^[15]^ The vector of the catheter can be used to determine whether there is contact by side between the catheter and the tissue, as direct contact can increase steam pop.^[17]^ In this study, one of the noted tamponades happened because of direct contact between the catheter tip against the antrum during ablation, which would suggest that steam pop and tamponades can be avoided when the catheter tip contacts the tissue within an adequate force range and contacts it by side during ablation. The rarity of pop incidents indicates that this ablation technique was safe and repeatable.

### 4.3 The 3D system improves effectiveness of the ablation procedure

The CARTO 3 system can be used to visualize the catheter within the 3D shell of LA in real-time, providing more information about the structure of the LA than x-rays.^[14]^ Both the apex and the front part of the catheter are visible on the screen, and the vector of the apex can be used to clearly reposition the catheter. Accurate positioning of PV antrum was a key component in green ablation. Combining the 3 characteristics, with particular emphasis on the noted drop-off motion, the location of PV antrum can be positively determined. All of these movements can be visualized on the CARTO screen, enabling PVI without using x-rays. Continuous catheter movement during ablation results in fewer or no gaps and, when combined with optimized catheter CF, this movement improves the outcome of PVI for PAF.^[18]^

The system is able to automatically record each point of ablation around the 3D structure of the PV antrum as the CF and duration time were maximized, avoiding wasteful and excessive ablation duplication. This may explain the increased efficiency and success of RFCA with 3D system,^[19]^ as noted when RFCA has been used as a first-line strategy for PAF patients.^[2]^ In this study, the average procedure time was about 1 hour (∼65 minutes), and the ablation time was within 45 minutes (∼38 minutes). Moreover, the immediate success rate of PVI was 100%.

### 4.4 Study limitations

This study included a relatively small number of patients, which suggests that asymptomatic and low-frequency recurrence of AF could have been undetected in the duration of this study. The change in AF burden in patients with PAF was not quantified, but the ablation design for PVI was close to PV ostial ablation. Therefore, further studies with a larger number of patients or more intensive ECG follow-up are warranted. As permanent PVI and potentially proarrhythmic effects of inadvertent substrate modification are important to patient health, this type of randomized study is clinically relevant for patients with persistent AF.

## 5 Conclusions

Reconstruction LA and isolation PV ablation using single CF-assisted catheter without x-ray exposure was both safe and effective. CF was positively associated with effective outcomes and LA dimensions negatively with effective ones.
